# Re-ballooning of sealing frame for intraoperative paravalvular leak during rapid deployment aortic valve replacement: a report of two cases

**DOI:** 10.1186/s44215-025-00198-2

**Published:** 2025-03-10

**Authors:** Kentaro Yuda, Shintaro Katahira, Naoki Masaki, Tatsuya Tago, Kota Itagaki, Katsuhiro Hosoyama, Koki Ito, Yusuke Suzuki, Goro Takahashi, Kiichiro Kumagai, Yoshikatsu Saiki

**Affiliations:** Division of Cardiovascular Surgery, Tohoku University Graduate School of Medicine, Sendai, Miyagi 980-8574 Japan

**Keywords:** Rapid deployment aortic valve replacement, Paravalvular leak, Re-ballooning, Balloon aortic valvuloplasty

## Abstract

**Background:**

Rapid deployment aortic valve replacement (RDAVR) has been widely adopted, but concerns about postoperative paravalvular leak (PVL) associated with its use remain. PVL is linked to an increased risk of long-term mortality; however, there is no consensus on its treatment.

**Case presentation:**

Case 1: A 76-year-old female with severe aortic stenosis underwent RDAVR via median sternotomy. Intraoperative transesophageal echocardiography (TEE) revealed moderate PVL at the left-noncoronary cusp commissure. Three horizontal mattress stitches were applied from outside the aorta through the prosthetic sewing cuff to address the PVL site; however, the leak persisted. It was noted that the balloon-expandable sealing frame was slightly protruding inward at a location corresponding to the PVL site. Accordingly, balloon dilatation was performed under direct vision, and the PVL resolved. Postoperatively, no conduction disorders were observed. At the 24-month follow-up, echocardiography showed no recurrence of PVL.

Case 2: A 78-year-old male with severe aortic stenosis underwent RDAVR in a standardized fashion. Intraoperative TEE revealed moderate PVL at the right coronary cusp side. The balloon-expandable sealing frame was found not to have fully expand outward at the PVL site. Balloon dilatation was therefore performed as in Case 1, successfully resolving the PVL. No postoperative conduction disorder was encountered. At the 12-month follow-up, echocardiography revealed no recurrent PVL.

**Conclusions:**

Direct intraoperative re-ballooning is a potentially effective option for addressing intraoperatively identified PVL after RDAVR.

**Supplementary Information:**

The online version contains supplementary material available at 10.1186/s44215-025-00198-2.

## Background

Rapid-deployment aortic valve replacement (RDAVR) has been widely used to make surgery less invasive, reducing aortic cross-clamp time and facilitating its application in minimally invasive cardiac surgery [1]. This approach yields favorable results. However, the risk of postoperative paravalvular leak (PVL) is higher than that of conventional aortic valve replacement (AVR) [1]. Although some case reports have detailed the use of percutaneous balloon aortic valvuloplasty (BAV) for treatment of postoperative PVL [2], the efficacy of intraoperative balloon dilatation of the sealing frame under direct vision remains undefined in addressing intraoperative PVL. In this report, we present two cases successfully managed using intraoperative re-ballooning under direct vision.

## Case presentation

### Case 1

**Fig. 1:**
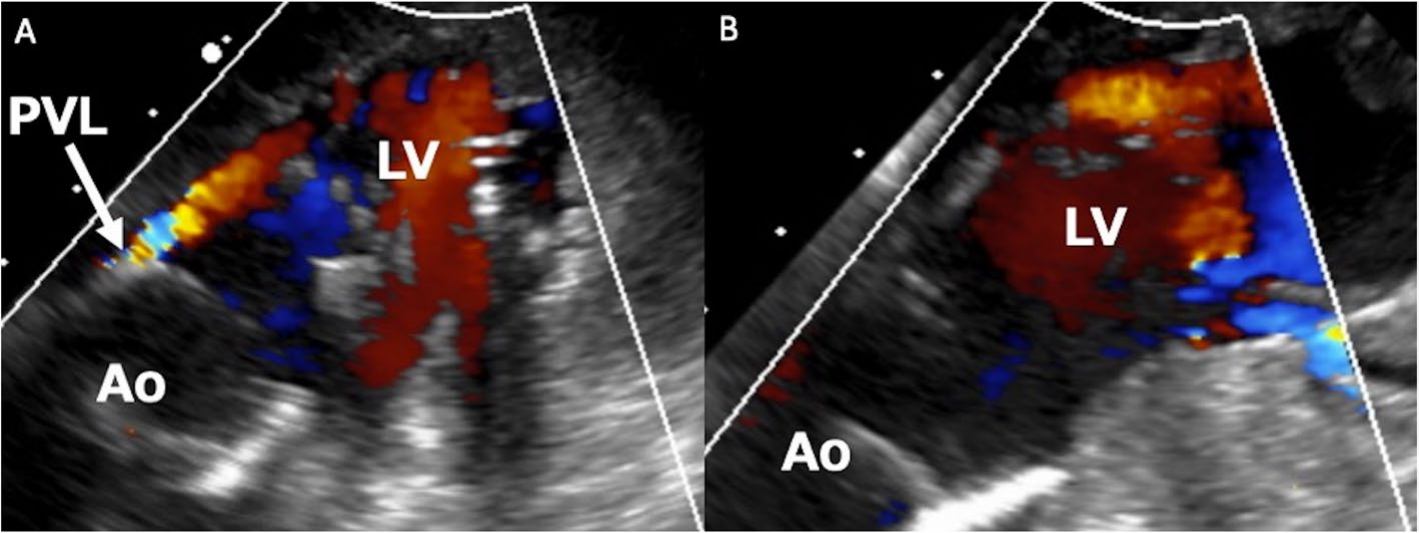
Intraoperative TEE in Case 1. Intraoperative transesophageal echocardiography in Case 1 revealed (**A)** a moderate paravalvular leak at the left-noncoronary commissure side, and **B** the leakage resolved after re-ballooning the sealing frame

**Fig. 2:**
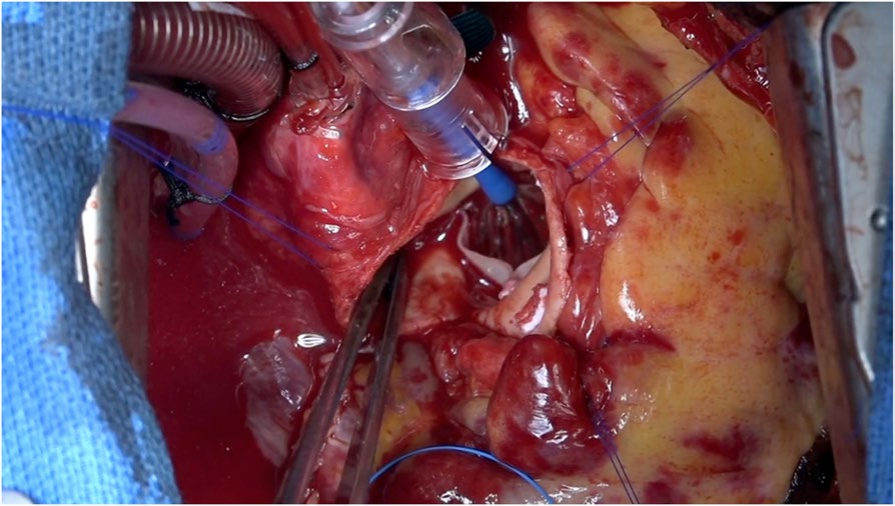
Surgical view of direct re-ballooning. Direct balloon dilatation was performed using the originally incorporated balloon, with the same dilatation pressure and duration as in the initial balloon dilatation

A 76-year-old female with severe aortic stenosis underwent transthoracic echocardiography, showing an aortic annular diameter of 21 mm and left ventricular outflow tract (LVOT) diameter of 21 mm. RDAVR was performed via median sternotomy. The aortic cusps were resected, and the annulus was adequately decalcified using a Cavitron ultrasonic surgical aspirator. A 21-mm EDWARDS INTUITY Elite valve (Edwards Lifesciences, Irvine, CA, USA) was implanted and expanded with a balloon at 4.5 atm for 10 s. As we approached weaning from the cardiopulmonary bypass, transesophageal echocardiography (TEE) revealed moderate PVL at the left-noncoronary commissure side (Fig. 1A). The aorta was re-clamped, and three horizontal pledgetted mattress stitches were applied from outside the aorta through the prosthetic sewing cuff to address the PVL site. However, the leak persisted after reperfusion of the heart. A third aortic clamping was performed. We realized that the balloon-expandable sealing frame was slightly protruding inward at a location corresponding to the PVL site. Accordingly, balloon dilatation was performed at 4.5 atm for 10 s under direct vision, which resulted in the resolution of PVL (Figs. 1B and 2). An additional movie file shows these consequences in more detail (see Additional file 1). Postoperatively, no conduction disorders were observed. At the 24-month follow-up, echocardiography revealed no PVL recurrence.

### Case 2

**Fig. 3:**
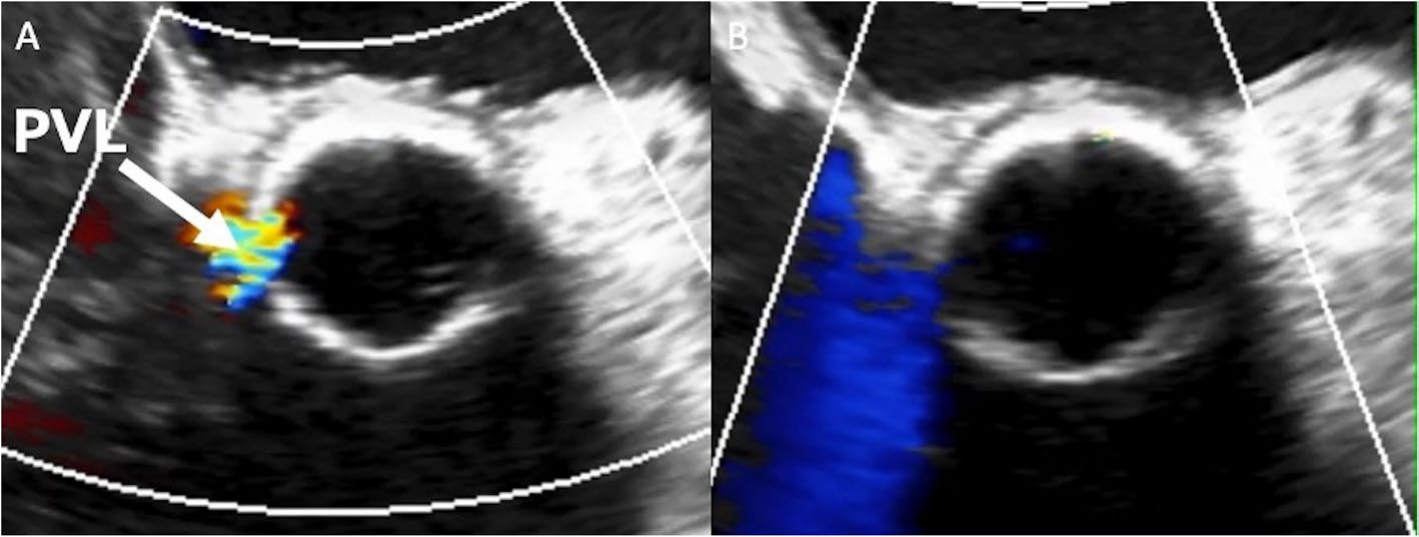
Intraoperative TEE in Case 2. In Case 2, intraoperative transesophageal echocardiography showed (**A**) a moderate paravalvular leak at the right coronary cusp side, and **B** the leakage resolved after re-ballooning the sealing frame

A 78-year-old male with severe aortic stenosis underwent a 21 -mm Intuity valve implantation, similar to Case 1. Intraoperative TEE revealed moderate PVL at the right coronary cusp side (Fig. 3A). The aorta was re-clamped, and three horizontal pledgetted mattress stitches were applied from outside the aorta through the prosthetic sewing cuff at the PVL site. We identified that the balloon-expandable sealing frame did not fully expand outward at the location corresponding to the PVL site. Thus, balloon dilatation was performed at 4.5 atm for 10 s under direct vision, and eventually, the PVL resolved (Fig. 3B). There was no postoperative conduction disorder. At the 12-month follow-up, echocardiography revealed no recurrence of PVL.

In both cases, we used the originally incorporated balloon with the same dilatation pressure and duration as in the initial balloon dilatation. Moreover, no apparent complications related to this technique, including conduction disorders, were observed.

## Discussion

Between February 2020 and December 2024, our institution performed 53 cases of RDAVR using the Intuity valve. Intraoperatively, moderate or greater PVL was observed in two cases (3.8%), which are presented in this report. Postoperatively, trivial PVL was observed in six cases, mild PVL in two cases, and moderate PVL in one case. One patient with postoperative moderate PVL experienced progression to severe PVL, leading to worsening heart failure, which necessitated BAV.

There are some concerns regarding PVL in RDAVR. In cases treated with transcatheter aortic valve implantation, even mild PVL is associated with an increased risk of long-term mortality [3]. In the context of RDAVR, residual PVL can adversely affect the prognosis, and timely intervention for identified PVL is imperative.

The primary cause of PVL in conventional AVR is inadequate fitting of the prosthetic valve cuff to the aortic annulus, often due to uneven stitches or tissue disruption. Additional stitches may potentially resolve such PVLs. By contrast, RDAVR does not involve circumferential sewing of the prosthetic valve cuff to the annulus. Some instances of PVL in RDAVR may arise from insufficient initial balloon expansion, leading to inadequate fitting of the sealing frame to LVOT. In the reported two cases, we observed the inner aspect of the balloon-expandable sealing frame during repeated cardiac arrest to identify the suboptimally expanded area corresponding to the PVL site. In such situations, adding some sutures on the sewing cuff at the PVL site may be less effective. By and large, residual calcification of the annulus may disturb the sealing frame fitting of LVOT during initial balloon dilatation. Thus, adequate decalcification of the annulus and precise sizing are crucial for preventing PVL after RDAVR [4]. Even with proper decalcification and sizing, intraoperative PVL may occur as observed in our two present cases. On this occasion, additional balloon dilation of the sealing frame under direct vision may be effective if PVL persists. In both cases, additional stitches were applied but proved ineffective, particularly in the first case. Closer inspection revealed insufficient expansion of the sealing frame in both cases, which was identified as the main cause of PVL. This issue was solved by re-ballooning, which we considered to be an immediately effective measure.

While there are case reports addressing “postoperative” PVL with percutaneous BAV [2], the treatment of “intraoperatively identified” PVL has not yet been fully established. Gennari et al. reported a case of intraoperative PVL that was successfully treated through direct re-ballooning using an off-label balloon [4]. However, they did not provide information on the appropriate balloon pressure or selection of balloon size. In our cases, we achieved favorable results using the originally incorporated balloon, with the same dilatation pressure and duration as in the initial balloon dilatation. In RDAVR, excessive dilatation increases the risk of conduction disturbance [5]. Re-ballooning with the initially used balloon proved effective, and excessive dilatation with a larger balloon should be avoided. Additionally, intraoperative percutaneous BAV, as another treatment option, could reduce the cross-clamp time; however, the procedure requires hybrid operating rooms equipped for cardiac angiography and fluoroscopy, incurring additional costs. Furthermore, BAV carries a potential risk of damage to the bioprosthetic valve leaflets due to balloon abrasion or commissural tears caused by excessive dilatation. In BAV via the percutaneous approach, selectively dilating the sealing frame while confirming its precise position is challenging. In contrast, direct vision re-ballooning is limited to the sealing frame under the sewing cuff, thereby reducing the risk of damaging the bioprosthetic valve leaflet. Additionally, intraoperative endoscopic confirmation to ensure full expansion of the sealing frame may help prevent PVL caused by inadequate fitting.

This presentation has several limitations. First, it includes only two cases, which is a small sample size. Additionally, while mid-term outcomes have been favorable, the long-term results remain unknown. Further follow-up and larger studies are warranted in the future.

In conclusion, direct intraoperative re-ballooning is a potentially effective option for addressing intraoperatively identified PVL after RDAVR.

## Supplementary Information

Additional file 1. Surgical video of performing direct re-ballooning. After the third aortic clamping, direct balloon dilatation of sealing frame was performed using the originally incorporated balloon, with the same dilatation pressure and duration as in the initial balloon dilatation, which resulted in the resolution of PVL.

## Data Availability

The data sets supporting the conclusions of this study are included within the article and its additional files.

## Abbreviations

RDAVR: Rapid deployment aortic valve replacement
PVL: Paravalvular leak
AVR: Aortic valve replacement
BAV: Balloon aortic valvuloplasty
LVOT: Left ventricular outflow tract
TEE: Transesophageal echocardiography
