# Supplementary material for: Re-ballooning of sealing frame for intraoperative paravalvular leak during rapid deployment aortic valve replacement: a report of two cases
Source: Gen Thorac Cardiovasc Surg Cases. 2025 Mar 10;4:13. doi: 10.1186/s44215-025-00198-2 (PMC11892181; doi:10.1186/s44215-025-00198-2)
Supplement: Supplementary file 1 — Additional file 1. Surgical video of performing direct re-ballooning. After the third aortic clamping, direct balloon dilatation of sealing frame was performed using the originally incorporated balloon, with the same dilatation pressure and duration as in the initial balloon dilatation, which resulted in the resolution of PVL. [file 44215_2025_198_MOESM1_ESM.pptx]

## Slide 1
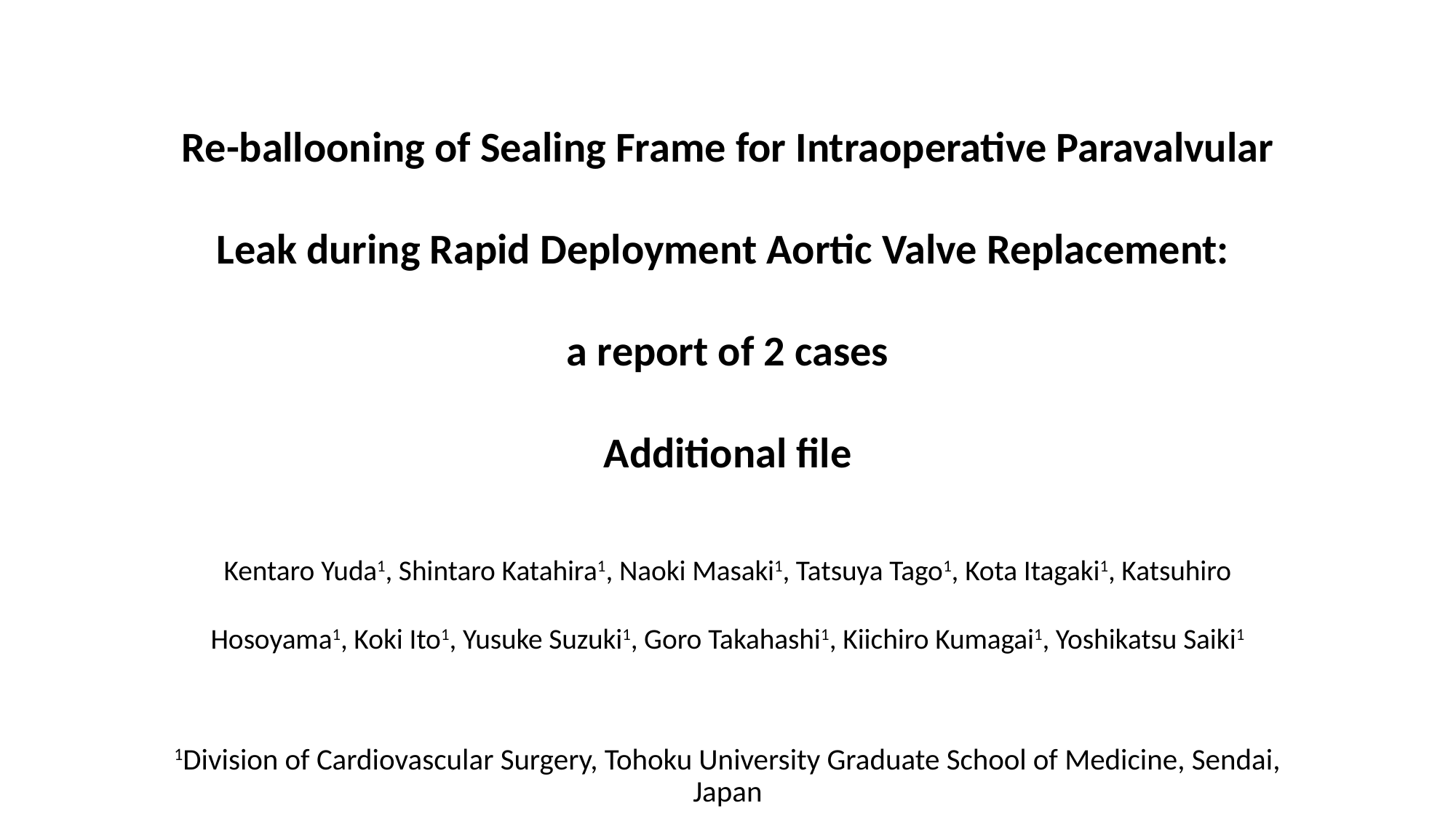

# Re-ballooning of Sealing Frame for Intraoperative Paravalvular Leak during Rapid Deployment Aortic Valve Replacement: a report of 2 cases Additional file
Kentaro Yuda1, Shintaro Katahira1, Naoki Masaki1, Tatsuya Tago1, Kota Itagaki1, Katsuhiro Hosoyama1, Koki Ito1, Yusuke Suzuki1, Goro Takahashi1, Kiichiro Kumagai1, Yoshikatsu Saiki1
1Division of Cardiovascular Surgery, Tohoku University Graduate School of Medicine, Sendai, Japan

## Slide 2
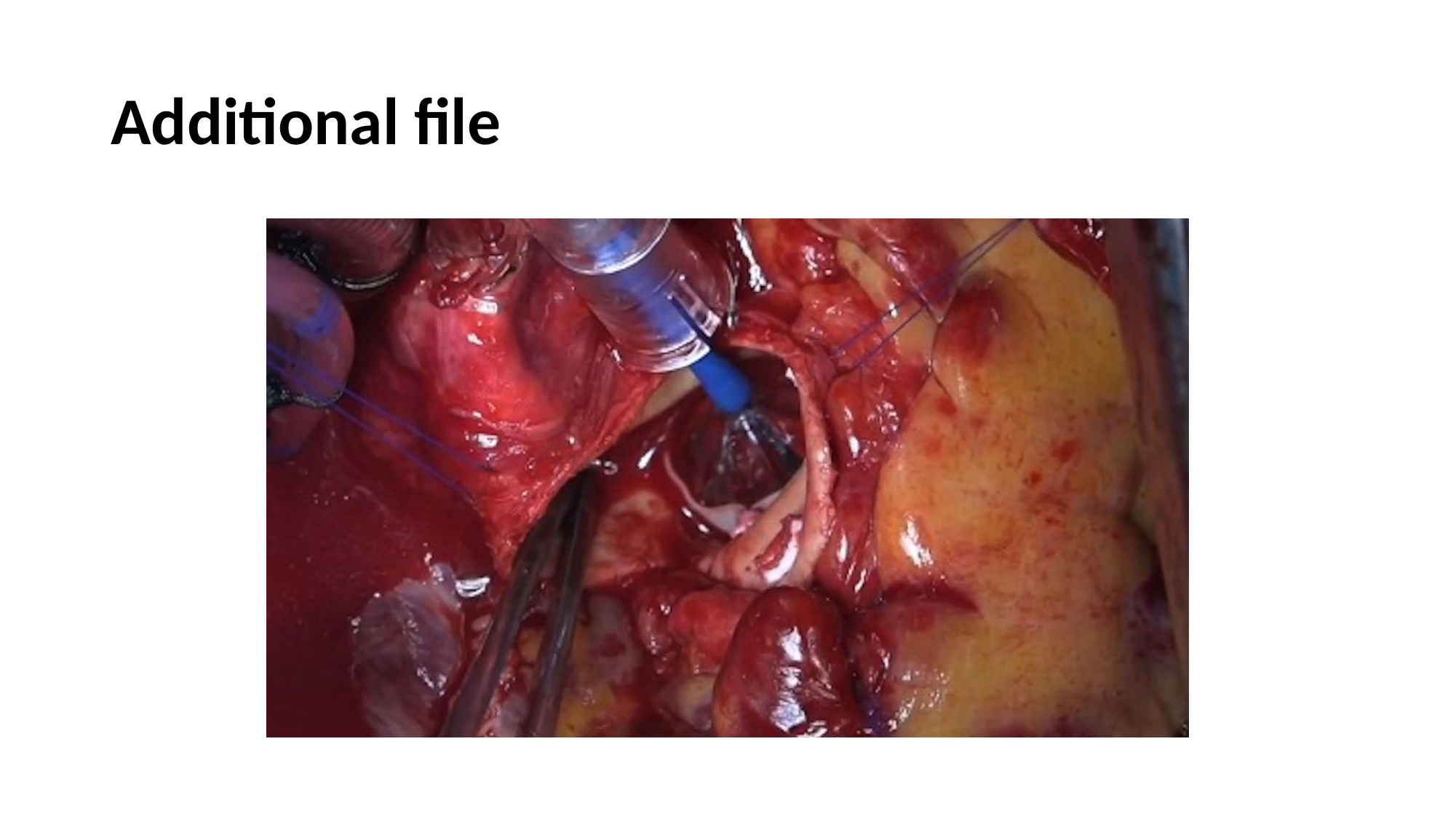

# Additional file
